# Augmenting Performance: A Systematic Review of Optical See-Through Head-Mounted Displays in Surgery

**DOI:** 10.3390/jimaging8070203

**Published:** 2022-07-20

**Authors:** Mitchell Doughty, Nilesh R. Ghugre, Graham A. Wright

**Affiliations:** 1 Department of Medical Biophysics, University of Toronto, Toronto, ON M5S 1A1, Canada; nilesh.ghugre@utoronto.ca (N.R.G.); graham.wright@sri.utoronto.ca (G.A.W.); 2 Schulich Heart Program, Sunnybrook Health Sciences Centre, Toronto, ON M4N 3M5, Canada; 3 Physical Sciences Platform, Sunnybrook Research Institute, Toronto, ON M4N 3M5, Canada

**Keywords:** augmented reality, head-mounted displays, surgical navigation, medical imaging, human factors

## Abstract

We conducted a systematic review of recent literature to understand the current challenges in the use of optical see-through head-mounted displays (OST-HMDs) for augmented reality (AR) assisted surgery. Using Google Scholar, 57 relevant articles from 1 January 2021 through 18 March 2022 were identified. Selected articles were then categorized based on a taxonomy that described the required components of an effective AR-based navigation system: data, processing, overlay, view, and validation. Our findings indicated a focus on orthopedic (n=20) and maxillofacial surgeries (n=8). For preoperative input data, computed tomography (CT) (n=34), and surface rendered models (n=39) were most commonly used to represent image information. Virtual content was commonly directly superimposed with the target site (n=47); this was achieved by surface tracking of fiducials (n=30), external tracking (n=16), or manual placement (n=11). Microsoft HoloLens devices (n=24 in 2021, n=7 in 2022) were the most frequently used OST-HMDs; gestures and/or voice (n=32) served as the preferred interaction paradigm. Though promising system accuracy in the order of 2–5 mm has been demonstrated in phantom models, several human factors and technical challenges—perception, ease of use, context, interaction, and occlusion—remain to be addressed prior to widespread adoption of OST-HMD led surgical navigation.

## 1. Introduction

**Figure 1:**
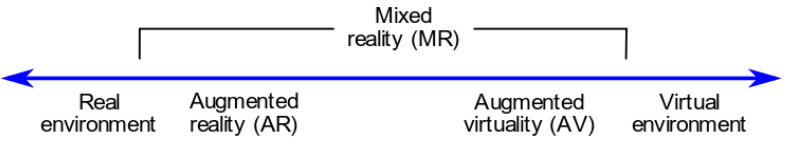
The continuum proposed by Milgram and Kishino [1] describing the interactions between reality and virtuality in creating augmented reality experiences. Reproduced without modification from Wikimedia Commons (source: https://commons.wikimedia.org/wiki/File:Virtuality_continuum_2-en.svg, (accessed on 23 March 2022) licensed under: https://creativecommons.org/licenses/by-sa/4.0/deed.en (accessed on accessed on 23 March 2022).

In their 1994 paper, Milgram and Kishino detail a continuum to describe the ways in which virtual and real environments can be combined to create different experiences for a user [1]. The left side of the spectrum in Figure 1 describes a fully real environment with no virtual elements; conversely, the right side of the spectrum details an environment which consists of an immersive, fully virtual experience, where a user can interact with synthetic elements. Augmented reality (AR) encompasses a combination of real world and virtual components and is the focus of this proposed work (Figure 1). Extended reality (XR) is a recently coined term used to broadly describe immersive technologies, such as AR, virtual reality (VR), and mixed reality, under a single umbrella.

### 1.1. Medical Augmented Reality

Augmented reality led guidance for surgical navigation was first suggested nearly 40 years ago, where Kelly et al. (1982) superimposed tumor outlines from preoperative CT data into the view of a surgical microscope rigidly attached to a stereotactic frame [2]. Though the accuracy of this initial system was not acceptable for clinical deployment, the contributions from this work inspired future investigations into frameless stereotaxy (or image-guided surgery) in neurosurgery [3], and today, image-guided surgery is routinely used for treatment of a multitude of brain disorders.

#### Surgical Navigation Strategies

In image-guided surgery, virtual content is typically generated from preoperative or intraoperative medical imaging data and visualized adjacent to the surgical scene on a monitor, or, in the case of an optical see-through head-mounted display (OST-HMD), projected directly onto the surgical scene [4]. Errors in registration, calibration, and latency due to tracking and rendering must be minimized to ensure that virtual 3D models precisely represent the current patient-specific anatomy. Surgical navigation systems are essential to the success of many modern surgical interventions, due to their capacity for continuous and precise intraoperative localization of surgical tools and tissue with respect to the patient.

Although there have been significant efforts into the design of OST-HMD led surgical navigation platforms, applications have remained constrained to research lab environments and have experienced little clinical uptake [5,6]. As evidenced by the issues raised in recent literature, the poor clinical uptake of OST-HMDs for surgical guidance can be partially attributed to a lack of HMD performance [7] and rendering resolution [8]; however, perceptual challenges [9,10], surgical context and interaction limitations [11,12], and registration and occlusion challenges [13,14] remain the key hurdles to the widespread clinical adoption of these technologies.

For the effective and clinically successful adoption of OST-HMD based AR guidance, we believe that there is a requirement to: (1) address the perceptual and human factors limitations regarding optimal modes of virtual content visualization and information display; (2) reduce the interaction burden on surgeons required to manually adapt the appearance and presentation of critical guidance information, in the form of virtually augmented entities, to their current surgical context; and (3) minimize the setup time and potential for user-introduced error during the preparation and calibration of a navigation solution for surgical tracking. We hope that the detailed analysis and commentary introduced in this review paper will serve to highlight strategies for mitigating the impact of these challenges, furthering the likelihood of clinically successful OST-HMD led surgical navigation in the future.

## 2. Background

In the context of AR visualization, displays can be classified into three separate categories: hand-held displays, spatial display systems, and head-mounted displays (HMDs), based on their position between the viewer and the real environment [15]. A fundamental requirement of an AR system is the ability to combine, register, and display interactive 3D virtual content with real-world scenes in real time [15]. Although the focus here is on HMDs, we also briefly introduce handheld and spatial display systems for context [8,13,15,16].

### 2.1. Handheld and Spatial Displays

Hand-held displays include video see-through displays like mobile devices (smartphones or tablets) which are held near the user. Hand-held AR has seen broad uptake into applications in entertainment, product marketing, education, and social networking [17]. With the current computing power of smartphone hardware, hand-held AR experiences can be effectively delivered to the vast user base of billions of device owners; however, the ergonomic limitations of mobile AR, such as the small screen size for augmenting virtual content and requirement for hand-held interaction, limit the effective use cases [17]. Spatial display systems are placed statically within an environment and include screen-based video see-through displays and projective displays [15]. Video see-through spatial display systems, such as conventional 2D and 3D monitors or televisions, blend virtual content with real imagery for user consumption. A limitation of spatial AR is the static nature of the display and, due to remote viewing, the requirement for a user to create a mental mapping to understand the context and placement of virtually augmented information shown on the display when mapped in the real world [18].

### 2.2. See-Through Head-Mounted Displays

**Figure 2:**
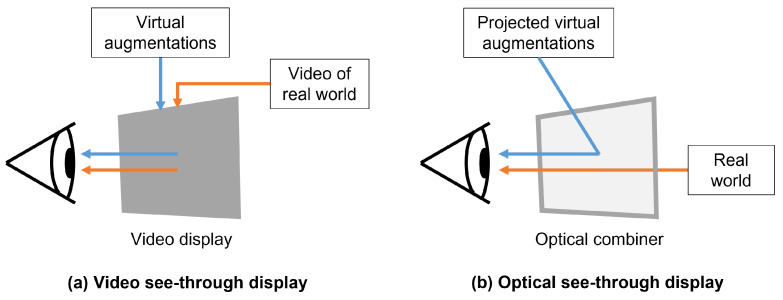
A simplified image representation of video see-through (**a**) and optical see-through (**b**) head-mounted displays (HMDs). Video see-through HMDs use an opaque video display to present virtual content combined with video of the real world. Real world video is typically captured by front-facing red-green-blue cameras on the front of the HMD. Optical see-through HMDs make use of a transparent optical combiner to merge virtual content, projected into the field of view of the wearer, with a view of the real world.

See-through HMDs provide hands-free interaction, direct projection of virtually augmented entities into the field of view (FOV) of the user, and 3D viewing capacity via stereoscopic rendering. See-through HMDs can be further classified into video see-through HMD (Figure 2a) and OST-HMD (Figure 2b) categories based on their mechanism of display [19]. Video see-through HMDs make use of front-facing cameras to record and digitize the scene before combining with computer-generated virtual images, limiting the user view to the camera field-of-view and visual display resolutions, and potentially introducing lag [16] and geometric aberrations, such as distorted spatial perception [20]. Recent work has investigated the use of non-orthostereoscopic video see-through HMDs to minimize the contribution of these geometric aberrations to perceptual error [21]. Optical see-through HMDs are less obtrusive to the user and allow for an unhindered view of reality and natural stereo vision with no additional disruption to their view due to lag or reduced resolution. Optical see-through HMDs make use of beam splitting holographic optical elements to visualize computer generated graphics [19], although, they have FOV and contrast limitations due to the quality of display technology and require additional calibration to accommodate for the eye positions of an individual user [22]. Additionally, there are challenges with occlusion in OST-HMDs, where distant virtual content will appear on top of nearer real objects [23]. For uses in image guidance for safety-critical applications, optical see-through systems have shown a clear benefit over video see-through systems due to the ability of the wearer to maintain an uninterrupted and instantaneous view of the scene [24,25]. In this review, we focus specifically on the evaluation of optical see-through HMDs.

#### Optical See-Through Head-Mounted Displays

Optical see-through HMDs were developed and first introduced in the 1960s, with Ivan Sutherland demonstrating the first computer graphics-based HMD in 1968 using miniaturized cathode-ray tubes as stereoscopic displays and a mechanical tracker to provide head position and orientation in real-time [26]. Though there have been significant recent improvements to HMD technology, the basic display configuration of OST-HMDs remains relatively unchanged from the designs of the early 2000s which used half-silvered mirrors or beam combiners to merge virtual content with the real view [27]. These OST-HMDs are operated by rendering virtual content on a 2D micro display outside the FOV of a user and redirecting light rays to the eye of the wearer using a beam combiner [10]. Lenses are placed between the beam combiner and display to focus the virtual images on a semitransparent surface of projection (SSP) at a viewing distance that is comfortable for the wearer, allowing the wearer to perceive 3D virtual augmentations through stereoscopic vision of the two 2D SSPs [28].

### 2.3. Overview of Commercially Available Optical See-Through Head-Mounted Displays

**Figure 3:**
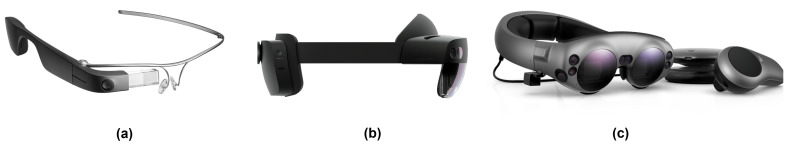
Images of the prominent commercially available optical see-through head-mounted displays. (**a**) The Google Glass 2 Enterprise Edition with on-board computing (Google, Mountain View, CA, USA). (**b**) The Microsoft HoloLens 2 headset with on-board computing (Microsoft, Redmond, WA, USA). (**c**) The Magic Leap 1 headset with a separate computing pad and controllers (Magic Leap, Plantation, FL, USA).

Coinciding with the ongoing COVID-19 pandemic and global paradigm shift to remote work, there have been significant investments by technology companies, including Google, Apple, Microsoft, and Meta (Facebook), into AR and VR HMD technology in the creation of their own renditions of 3D virtual worlds to enable enhanced social connection and telepresence (metaverse). We focus solely on outlining the relevant commercially available OST-HMDs and do not consider video see-through HMDs and virtual reality HMDs. Images of the relevant commercially available OST-HMDs are included in Figure 3. A detailed summary of the relevant OST-HMDs and their associated technical specifications are included in Table 1.

As indicated in Table 1, all the catalogued OST-HMDs have six-degrees of freedom (6DoF) simultaneous localization and mapping (SLAM) capabilities. Simultaneous localization and mapping combines multiple sensor inputs to create, and continuously update, a construction of an unknown environment and allow for the HMD to know its position and orientation (pose) within the environment. With continual knowledge of HMD pose, it becomes possible to place or anchor virtually augmented entities to locations in the real world and create an immersive virtual experience.

#### Virtual Model Alignment

**Figure 4:**
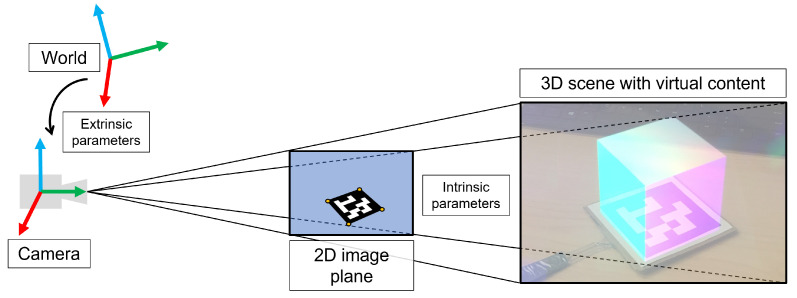
Depiction of the fundamentals of marker-based tracking. The intrinsic camera parameters serve to project three-dimensional (3D) content in the camera coordinate system to its two-dimensional (2D) representation on the camera image plane by perspective projection. The extrinsic parameters relate the position and orientation of the world coordinate frame to the camera coordinate frame. Combined, the intrinsic and extrinsic camera parameters allow the relation of 3D points in the world to 2D points on the camera image plane and enable marker-based tracking and precise augmentation of virtual content.

A foundational aspect of AR is the capacity for accurate spatial alignment of virtual content with real objects in the world [8]. Conventional strategies for HMD-led tracking of real objects rely on 2D feature-matching of square marker fiducials captured through input video from a red-green-blue (RGB) camera (Figure 4).

Sensor calibration and estimation of the intrinsic and extrinsic parameters of an RGB camera is a fundamental requirement prior to performing any sort of computer vision-based task, as it enables the relation of points in the world coordinate frame to their respective image projections on the camera plane through perspective projection [29] (Figure 4). The intrinsic parameters (focal length, principal points, and skew) are computed once for a specific RGB camera using a planar chessboard-based calibration procedure [30]. The extrinsic parameters (rotation and translation) are constant provided the camera coordinate frame does not change with respect to the world.

In most cases, however, the wearer of the HMD will be moving around the environment, so the camera coordinate frame will be constantly changing with respect to the world, requiring the extrinsic parameters to be recomputed each frame; this is where marker tracking is of value, as it allows the precise localization of the HMD (and camera coordinate frame) relative to the markers through knowledge of marker size and the use of our intrinsic parameters that defined a projection matrix [31].

Simultaneous localization and mapping allows for HMD localization within the world; however, it does not provide the same accuracy as marker-based localization and is subject to drift caused by error propagation over time [32]. Though the accuracy and stability of SLAM is not enough to rely on for precise tasks, it can be invaluable as an alternative method to maintain alignment of virtual entities to real objects during instances where marker-based localization is unstable or lost [33].

### 2.4. Augmented Reality Perception

Although there have been significant improvements to the performance and comfort of OST-HMDs, limitations associated with human perception remain to be addressed. In the context of this review, perception refers to the quantitative assessment of a user’s interpretation and understanding of virtually augmented elements. Primary contributors to perceptual limitations of current OST-HMDs include: the small FOV of virtually augmented content; the obtrusiveness and weight of the device [34]; the low luminance of the micro-displays; the requirement for frequent recalibrations to maintain precise spatial alignment of virtual content [22]; depth perception and depth cues [35]; and the perceptual conflicts between the 3D view of the real world and the 2D virtual images on the stereoscopic lenses [10,36,37]. With the recent influx of commercial interest into HMD technology, we anticipate that the limitations due to device form factor, and challenges with FOV and display luminance will be addressed with upcoming device iterations. Instead, we will focus on a brief discussion of the other factors which contribute to perceptual challenges in commercially available OST-HMDs. Perhaps the most important is depth perception. Section 2.4.1 highlights visual cues and subsequent sections summarize how these are addressed by OST-HMDs.

#### 2.4.1. Depth Perception and Depth Cues

The human visual system relies on several sources of perceptual information related to depth, or depth cues, to perceive depth from imagery [35]. Cutting and Vishton have identified nine depth cues that are essential to depth perception; we will discuss the most prominent in detail: occlusion, binocular disparity, motion perspective, relative size/density, and vergence and accommodation [38]. Occlusion refers to the blocking of a distant object by a nearer one. Binocular disparity is the difference between image projections to left and right retinal images based on the horizontal separation of the eyes. Motion perspective describes the different inferred velocities of moving objects at different distances from the observer. Relative size refers to retinal angle of projection of similarly sized objects at different distances, where the farther object will project to a smaller retinal angle [35]. Accommodation refers to the changing shape of the human eye to bring objects into focus, and vergence describes the rotation of the left and right eyes to fixate on an object [9]. To fixate on a near object, the pupils converge and rotate towards each other. To fixate on a distant object, the pupils diverge and rotate away from each other. Binocular disparity and vergence and accommodation contribute to depth perception via physiological cues, whereas occlusion, motion perspective, and relative size/density rely on psychological cues [39]. Through the effective combination of these cues, it is possible to create imagery which will enable a user to perceive depth.

#### 2.4.2. Interpupillary Distance

To render a virtual scene on an OST-HMD, we require knowledge of the internal projection parameters of the eye cameras of the device. These internal projection parameters are typically provided by the manufacturer and will function to define the left and right eye virtual cameras. An additional metric, the interpupillary distance (IPD) of the user, is used to define the horizontal distance offset between the left and right virtual eye cameras to ensure comfortable 3D perception of virtually augmented content through binocular disparity of the 2D image pair. Early OST-HMDs included a predefined horizontal distance offset based on the average IPD of a typical user; however, this was not ideal as miscalibrations in the IPD can result in poor depth perception of the displayed virtual content [40]. Both the HoloLens and Magic Leap device classes include an application to estimate IPD which is launched when a new user wears the HMD. The second-generation HoloLens and Magic Leap headsets improve on the accuracy of IPD estimation of their predecessors using active-eye tracking. A secondary benefit of the active-eye tracking incorporated into second generation headsets is the capacity for adaptive correction for changes in the positioning of the HMD relative to the eyes of a wearer via additional corrections made to virtual content augmentation, reducing the contribution to distortion of virtual content [23].

#### 2.4.3. Vergence-Accommodation Conflict

Vergence-accommodation conflict is not unique to HMDs and is inherent to all conventional stereoscopic 3D displays which simulate 3D perception from a pair of 2D perspective images using binocular disparity and other depth cues [36]. To enable 3D perception of augmented virtual content, an OST-HMD requires an optical combiner that is positioned in front of the eye of a wearer to combine the optical path of the virtual display and real scene [36]. Vergence-accommodation conflict arises from the inability to render correct focus cues for the augmented virtual information which can appear at different distances from the corresponding virtual image plane [36,37] and can contribute to blurred content and visual fatigue during prolonged use [9]. In traditional stereoscopic HMDs, the vergence distance varies based on the depth of targets (virtual or real), whereas the accommodation, or focal distance, is fixed at the distance of the focal plane of the virtual display [9].

Depth of focus (*F*) refers to the range of distances in display space within which an image appears sharp and correctly formed and is measured in diopters (D), a standard depth of focus estimate is on the order of ±0.5 D [10,41]. Related to depth of focus is depth of field (DOF): the depth interval over which a stimulus remains in focus and the accommodative response of a viewer does not substantially change [41]. Depth of field is inversely related to depth of focus as DOF=1/F [41]. For a given fixation distance (*P*), we can estimate the near (${\mathrm{DOF}}_{\mathrm{near}}$) and far (${\mathrm{DOF}}_{\mathrm{far}}$) DOF as [41]:

(1) $${\mathrm{DOF}}_{\mathrm{near}}=\frac{1}{(1/P)+F};{\mathrm{DOF}}_{\mathrm{far}}=\frac{1}{(1/P)-F}.$$

As described earlier, the HoloLens 1 and 2 HMDs include a single fixed focal plane, set at a distance of 1.5–2 m [42,43]. In contrast, the Magic Leap 1 HMD includes two fixed focal planes, one for content near the user at a distance of 1 m and a second for room scale content at a distance of 3 m. With Magic Leap devices, the active focal plane is adaptively selected based on the rendering distance of virtual content. Multiple focal planes can contribute to reduced vergence-accommodation conflict; however, this occurs only when the virtual content appears within a threshold of the distance of the active focal plane. For example, with a focal plane distance of 1 m, we use Equation (1) to compute an estimated depth of field range from 0.67–2 m, meaning that within this threshold virtual content will appear without blur. Current commercially available OST-HMDs are not intended for augmenting virtual content in the peripersonal space (roughly 0.3–0.5 m from the wearer) and, as a consequence, can introduce discomfort and content blur for some users with long sessions.

## 3. Methods

### 3.1. Literature Search Strategy

A systematic literature review was conducted using Google Scholar and the following search terms (surgery “head mounted display” AND “augmented reality” OR “mixed reality” AND “optical see through” OR “hololens” OR “magic leap” OR “google glass”). The literature search was performed on 18 March 2022, and included research articles from 1 January 2021 through the current date.

**Figure 5:**
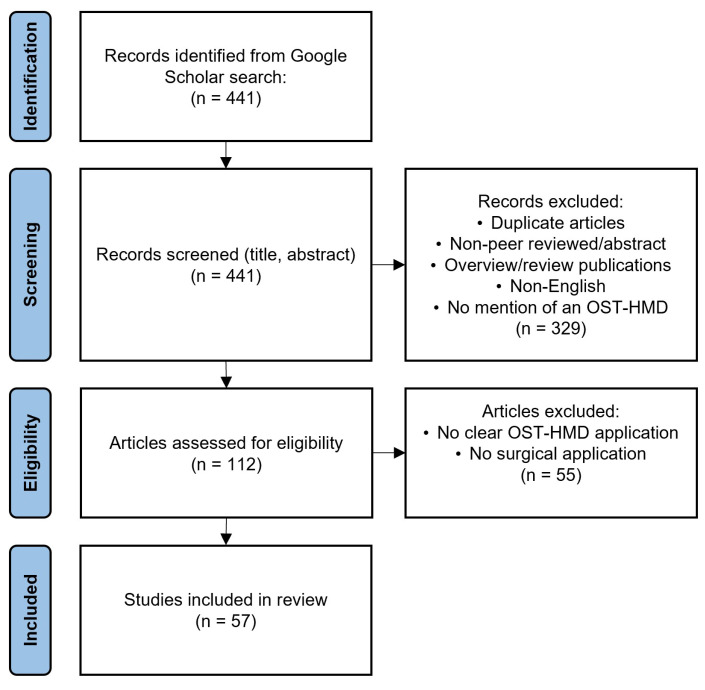
Search strategy of our systematic review.

The Google Scholar search resulted in 441 total records, from which we included 57 articles in the review. Duplicate articles, non-peer reviewed manuscripts, and work that did not: (1) describe the use of an OST-HMD; (2) indicate a focus on a surgical application; and (3) investigate the application of an OST-HMD in a surgical setting were removed. Further details of our systematic review search strategy are included in Figure 5.

**Figure 6:**
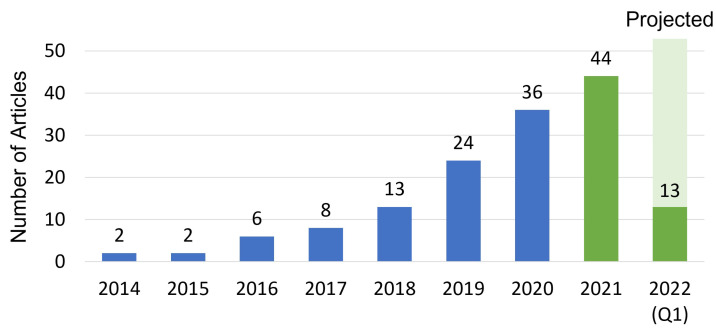
Article distribution per year of the conducted literature review from 2014 to 2022 (Q1). We have included the count provided by Birlo et al. for the years 2014 to 2020 [6] in blue with our contributions in green. As the review was conducted in the first quarter of 2022, an estimate of the full-year article publication count was arrived at by multiplying the count by four.

In Figure 6, we include a chart depicting the article frequency categorized by the publication year. For the years 2014 to 2020, we include the data provided in a recent review of OST-HMD indications in surgery by Birlo et al. [6]. In our review, we used similar search terms and criteria as Birlo et al. to provide comparable data. An uptick in the frequency of publications involving the use of OST-HMDs for surgical applications is visible and coincides with the introduction of commercially available HMDs: the first edition Google Glass OST-HMD in 2014 and the Microsoft HoloLens 1 OST-HMD in 2016.

### 3.2. Review Strategy and Taxonomy

**Figure 7:**
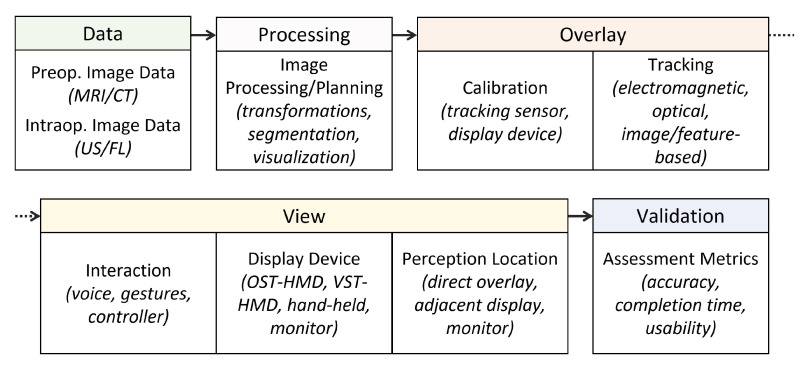
A taxonomy of the required components of an effective augmented reality based navigation solution for image guided surgery. Preoperative (preop.) data, such as magnetic resonance imaging (MRI) and computed tomography (CT), along with intraoperative (intraop.) data, such as ultrasound (US) and fluoroscopy (FL), are indicated. In the view category, the display device for visualization of virtual content is included, these could be optical see-through head-mounted displays (OST-HMDs) or video see-through head-mounted displays (VST-HMDs) for example. Our taxonomy is modified from the description provided by Kersten-Oertel et al. [44].

In Figure 7, we detail a taxonomy of the required components of an effective AR-based navigation solution for image guided surgery. Our taxonomy is adapted from the Data, Visualization processing, View (DVV) framework presented by Kersten-Oertel et al. [44]. Core requirements include preoperative image data (data); image processing (processing); calibration and tracking for augmented content overlay (overlay); interaction, display device, and perception location (view); and performance assessment metrics (validation).

## 4. Results and Discussion

### 4.1. Distribution of Relevant Articles by Surgical Application

**Figure 8:**
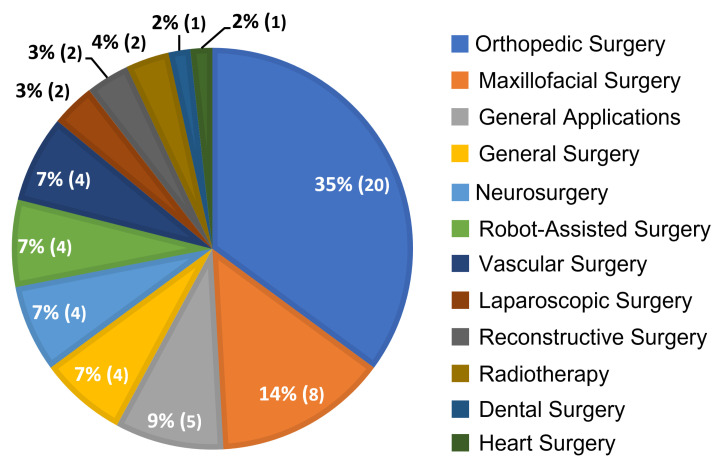
Distribution of the 57 articles included in the review based on the surgical speciality discussed.

In Figure 8, a breakdown of the 57 papers included in our survey is depicted based on their general surgical speciality. The majority of papers (n=20) focused on orthopedic applications, such as osteotomy [45,46,47,48,49] and K-wire placement [50,51], with the second largest (n=8) involving maxillofacial surgery procedures, such as tumor resection [52] and craniofacial fibrous dysplasia [53,54]. The prevalence of OST-HMD investigation in orthopedic and maxillofacial surgeries is likely due to the rigid nature of the relevant anatomy, the availability of consistent landmarks for registration and tracking, and the prevalence of commercially available surgical navigation suites.

General applications (n=5), general surgery (n=4), neurosurgery (n=4), robot-assisted surgery (n=4), and vascular surgery (n=4) were the next most prevalent. Robot-assisted surgery applications included orthopedic applications [55], general bedside tasks [56], and endoluminal interventions [57]. Papers focused on general surgery and associated applications included telementoring [58], teleproctoring [59], surgical navigation [60], and liver resection [61]. Neurosurgery-related applications included incision planning [62,63], navigation [64,65], and complex craniotomies [66]. Vascular surgery related papers focused on use for percutaneous femoral artery access [67] and tumor puncture [68].

Laparoscopic surgery (n=2), reconstructive surgery (n=2), radiotherapy (n=2), dental surgery (n=1), and heart surgery (n=1) were the last categories surveyed. Reconstructive surgery included orbital floor reconstruction [69] and radiotherapy applications involved AR-based patient positioning [70,71]. Laparoscopic surgery applications included cholecystectomy [72] and general laparoscopic surgery [73]. Heart surgery related applications included guiding the targeted delivery of media to the surface of the infarcted heart in regenerative medicine [33]. Otolaryngology applications included median neck and brachial cyst excision [74] and neck cancer [75].

### 4.2. Data

In Table 2, the articles are categorized based on the data type used for surgical guidance.

#### 4.2.1. Preoperative Image Data

The majority of papers made use of preoperative CT data as the input to the surgical guidance strategy, corresponding with the frequent application space of orthopedic surgery and the enhanced ability of CT to generate geometrically accurate representations of rigid anatomical structures, such as bone [45,76,77]. The second most frequently used data type was MRI, either as a standalone data type [62,72,78] or in combination with CT [49,52,53]. Magnetic resonance imaging has better sensitivity than CT when imaging soft tissue structures; however, it is less geometrically precise [79] and has less signal intensity when imaging bony structures. The registration of MRI and CT data is advantageous in situations where geometrically precise bone imaging and soft tissue information are used in tandem. Prerecorded video content was used in the case of an immersive telementoring solution to support the remote surgeon during a simulated task [58].

#### 4.2.2. Intraoperative Image Data

The most frequently used intraoperative data strategy was X-ray fluoroscopy, which is commonly used in orthopedic procedures as a real-time 2D imaging strategy to confirm the current navigation precision prior to performing a surgical action [57,80,81]. Ultrasound imaging was the second most frequently used form of intraoperative data, and is commonly used as a tool for confirming real-time tissue targets in needle biopsy procedures [82,83]. Telestrations, such as virtual arrows and other virtual annotations were common in telementoring and teleproctoring [58,59,60]. Intraoperative endoscope video is the primary real-time data source for laparoscopic and endoscopic procedures and robot-assisted surgery [56,82]. Cone-beam CT employs a similar imaging strategy as CT and improves on some limitations of X-ray fluoroscopy, mainly the 2D nature of the data, by providing the capacity for a 3D snapshot of the intraoperative patient anatomy [84,85]. The final intraoperative data sources were from patient sensors and monitoring equipment [86] and simulated intraoperative data [73].

### 4.3. Processing

Table 3 lists the paper distribution based on the type of processing to prepare the preoperative or intraoperative data for display on the OST-HMD. The most frequent type of preprocessing of preoperative data was the creation of surface rendered models (shells).

#### Image Data Processing

After preoperative imaging, Digital Imaging and Communications in Medicine (DICOM) formatted data are typically provided which can be read in an open source library, such as 3D Slicer (https://www.slicer.org/, accessed on 23 March 2022) [46,65,74,87], or proprietary software, such as Mimics (https://www.materialise.com/, accessed on 23 March 2022) [47,61,88] or Brainlab (https://www.brainlab.com/digital-o-r/surgical-planning/, accessed on 23 March 2022) [67,84]. Surface rendered models provide enormous utility due to their ease of creation, lightweight model representation, and additional modifiable visualization properties, such as color, transparency, wireframe representations, and heatmaps [89]. For performance limited devices, such as current commercially available OST-HMDs, the number of vertices used to represent a 3D virtual model needs to be limited to ensure a performant application; many groups have mentioned the simplification of their model using open source tools, such as Blender (https://www.blender.org/, accessed on 23 March 2022) and MeshLab (https://www.meshlab.net/, accessed on 23 March 2022) [61].

Other 3D information that was included alongside the surface rendered models included relevant preoperative planning information, such as locations of pedicle screws [47], target anatomical contours in maxillofacial surgery [54,90], and tissue deformation models in liver resection surgery [61]. Several papers mentioned the streaming of raw 3D data directly to the OST-HMD for visualization, including intraoperative stereo endoscope video for robotic bedside task support [56], flexible endoscope steering [91], and endoluminal interventions [57]. Unlike 3D surface models, volume-rendered 3D models do not require manual segmentation and preprocessing to create and instead rely on discrete sampling of a 3D dataset. Volume rendering requires significant memory and computing power for loading and performant data display, which likely has limited the uptake into OST-HMD-led surgical navigation. Several groups reported the use of volume-rendered models for display on an OST-HMD during guidance for use in maxillofacial tumor resection [52] and neurosurgery [64]. Some groups mentioned the use of 3D printed models created from segmented surface models of preoperative patient anatomy for enhanced visualization and assessment alongside the virtual model display [51].

Not all virtually augmented content needs to be inherently 3D for it to provide value during surgical navigation. Several groups reported the use of 2D information in the form of telestrations for telementoring in general surgical task training [58] or teleproctoring for assistance during neurovascular procedures [59]. Other uses of 2D data included the display of raw 2D ultrasound data for general intraprocedural guidance [83,92] and assistance during cervical pedicle screw placement [93].

### 4.4. Overlay

In Table 4, each paper is categorized based on the method of achieving virtual content overlay, using either an external tracker, the OST-HMD camera (RGB or infrared), or manual placement. Further, we include the tracking marker used to achieve this overlay: external markers, such as retroreflective spheres, electromagnetic instruments or typical visible markers; or optical markers, such as ArUco [31], Vuforia (https://developer.vuforia.com/, accessed on 23 March 2022), custom markers, retroreflective spheres, QR-codes, AprilTag [94], or a marker-less approach.

#### Tracking Strategies

The most frequently employed external tracking-led strategy involved the use of an infrared tracking sensor, particularly: the Northern Digital Inc., Polaris (https://www.ndigital.com/products/, accessed on 23 March 2022) and Optitrack (https://optitrack.com/, accessed on 23 March 2022) (Northern Digital Inc., Waterloo, ON, Canada), or Medtronic StealthStation (https://www.medtronic.com/ca-en/, accessed on 23 March 2022) (Medtronic, Minneapolis, MN, USA). The sensor was used to detect rigidly mounted retroreflective spheres on the OST-HMD and instruments. The infrared tracking sensor served to locate the OST-HMD and tracked tools relative to its frame of reference, enabling the precise overlay of virtual content, provided the user and tracked tools remain in direct and uninterrupted line-of-sight of the tracking sensor. Electromagnetic tracking is capable of removing the line-of-sight requirement by defining an electromagnetic field in which micro-sensors can be located; the most frequently used system was the Northern Digital Inc. Aurora (https://www.ndigital.com/products/, accessed on 23 March 2022). Electromagnetic tracking is accurate within a specified volume, however, it has difficulty maintaining precise tracking in the presence of metallic instruments or devices. An external stereo RGB camera sensor, the MicronTracker from ClaroNav (https://www.claronav.com/, accessed on 23 March 2022) (ClaroNav, Toronto, ON, Canada) was also explored as the primary tracking solution and requires the use of typical visible marker fiducials for tracking. External tracking using stereo RGB is subject to the same line-of-sight challenges as with infrared tracking. Overall, in the external tracking-led approach, the OST-HMD served as a display medium for existing surgical navigation suites or sensors [54,68,84,93,95], replacing the traditional 2D computer monitor as the primary method of visualization.

The most commonly reported OST-HMD-led tracking solution was surface tracking, involving the use of the RGB camera capabilities of the headset, and square marker fiducials, such as those used in the Vuforia [64,69,74,96] (PTC, Boston, MA, USA) or ArUco [31,46,77,91] libraries. There were several papers which reported the use of custom markers for surface-based tracking, though the performance did not significantly differ from ArUco or Vuforia tracking solutions [82,97]. In the surface-based tracking strategy, the square marker fiducials served to localize the patient and any tracked tools relative to the reference frame of the OST-HMD, allowing for the overlay of virtual content [33,46,88,91,92]. Other groups reported the use of retroreflective spheres and the infrared camera capabilities on the HoloLens 1, HoloLens 2, or custom devices for improved tracking accuracy and performance over monocular RGB led tracking [60,75,90,98]. Moving away from the reliance on constructed marker configurations, several papers reported the use of point-based marker-less tracking for virtual content overlay [48,50,76].

During OST-HMD-based tracking, the line-of-sight requirement still exists; however, the tracked surgical instruments and patient are more likely to remain in a similar view configuration from the point-of-view of the HMD, and the HMD only needs to localize the patient and tracked instrument reference frames. In the case of an external tracking system, the patient, tracked instruments, and surgeon wearing the HMD all need to remain in the field of view of the tracking sensor of the navigation system to maintain their relative reference frames. For OST-HMD-based overlay, the HoloLens 1 was the most frequently mentioned device, followed by the HoloLens 2 and Magic Leap 1. Several groups reported successful guidance based on the OST-HMD camera and surface registration with markers [46,47,74,86] and point registration based on feature matching [48,50,87,99]. The final overlay strategy discussed is manual placement of virtual content; in this case it was up to either the surgeon [72,81] or a secondary user [55,74] to ensure comfortable placement of virtual content. After placement, virtual content remained anchored in place based on the SLAM predictions included with the HMD.

### 4.5. View

In Table 5 the 57 papers included in this review are categorized by the view type employed for virtual content visualization. Categories include the type of interaction with virtual content, the display device used for visualization, and the perception location of virtually augmented elements.

#### 4.5.1. Interaction Paradigms

The most frequently utilized interaction paradigm was a combination of voice and gesture-based interaction (n=15) followed by control using gestures only (n=14) or voice only (n=3). A combination of voice and/or gesture based interaction was commonly used alongside the HoloLens 1 or HoloLens 2 OST-HMDs due to their out-of-the-box support for several gestures and ease of integration of new voice commands. The next most frequently utilized interaction paradigm included the use of a controller (n=3), which was common to papers which included the use of the Magic Leap 1 headset due to its reliance on controller-based manipulation of virtual content. Finally, the use of a Bluetooth keyboard and user head pose to control virtually augmented content was also reported.

#### 4.5.2. Display Devices

**Figure 9:**
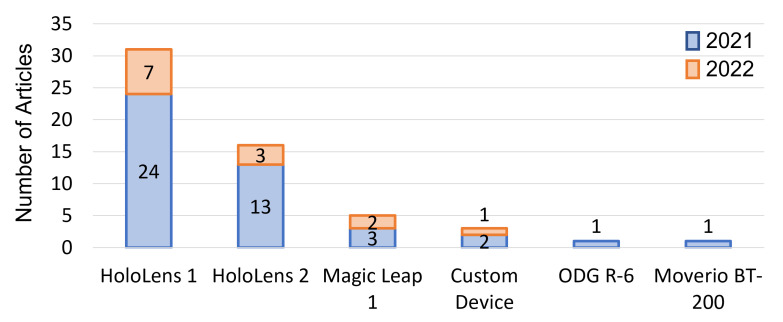
Distribution of the 57 articles included in the review based on the display device used.

Figure 9 includes a visual representation of the frequency of display device use; the HoloLens 1 was the most popular headset in 2021 (n=24) and in the first quarter of 2022 (n=7). The HoloLens 2 was the second most frequently used headset in 2021 (n=13) and in the first quarter of 2022 (n=3). The next most frequently used headset in 2021 and 2022 was the Magic Leap 1 (n=3) and (n=2), respectively. The ODG R-6, Epson Moverio BT-200, and other custom devices were less frequently reported.

#### 4.5.3. Perception Location

**Figure 10:**
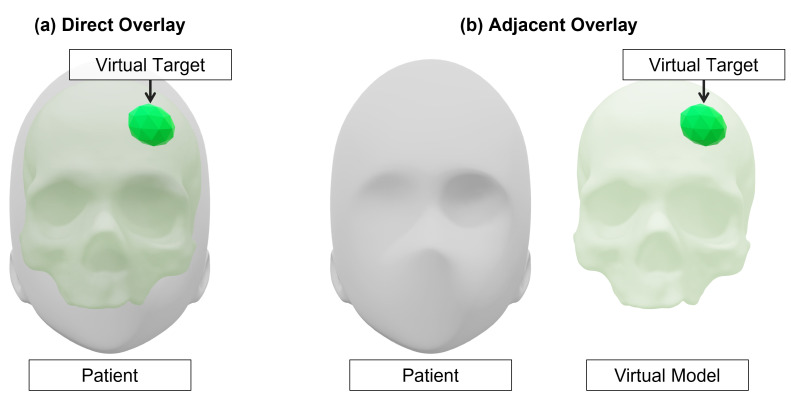
Demonstration of the difference between direct overlay of virtual content and adjacent overlay of virtual content. Direct overlay will include virtual content which is directly superimposed with the patient anatomy (**a**). Adjacent overlay involves the placement of virtual content next to the patient to improve data accessibility (**b**).

Perception location defined the placement of virtually augmented elements for surgical guidance; we defined two categories: direct overlay and adjacent overlay. Direct overlay involved the superimposition of virtual content with real objects, requiring a registration process for precise placement (Figure 10a). Adjacent overlay involved the placement of virtual content next to a real object and, as such, did not require the same stringent registration process for content alignment (Figure 10b). The majority of the surveyed articles (n=47) focused on direct overlay of virtual content with objects or tissue in the surgical scene to assist with guidance tasks. The remaining articles (n=10) involving adjacent overlay of virtual content centered on tasks which would typically rely on a computer monitor for data display and aimed to improve access to the relevant data. Use-cases involving adjacent overlay included the display of stereo endoscope video for robotic bedside assistants [56] and fluoroscopic imaging data [81] in the field of view of the user but adjacent to the patient.

### 4.6. Validation

A crucial aspect when evaluating any surgical navigation system involves validation: the assessment of quantitative performance metrics, such as accuracy and speed, and human factor considerations, such as perception, attention shift, and risk of error. In this section, the validation experiments performed in the surveyed articles are covered, and the main challenges and limitations described by the current literature are highlighted. In Table 6, the validation strategies employed by the articles included in the review are assessed and categorized based on the evaluation strategy, the reported accuracy metrics, and human factors limitations discussed.

#### 4.6.1. System Evaluation

The most common strategy for OST-HMD system evaluation included the use of an anthropomorphic phantom (n=37)—a reasonable approach, given the early stage investigational nature of many OST-HMD-based navigation platforms. Phantom models were used for assessment of error in orthopedic screw insertion [46,76,93], K-Wire placement for arthroplasty [50,51], maxillofacial drilling [54,69], head and neck cancer navigation [75], and as a means of simulating targeted media delivery in cardiac regenerative medicine [33]. Phantom models are relatively inexpensive and provide an excellent and safe platform for the initial quantitative assessment of a guidance system.

After phantom models, the next most frequently reported evaluation target was patients (n=16). Evaluation in patients primarily involved early feasibility assessments which included surgeon feedback and usability considerations, and typically the virtual information was not used to inform treatment decisions [46,62,63,72,96]. Several papers investigated the quantitative accuracy of OST-HMD led navigation in preliminary patient studies, including assessment of orthopedic pedicle screw navigation [47], neuronavigation [65], maxillofacial osteotomy tracing [90], and maxillofacial tumor resection [52].

Cadaver models were the next most commonly utilized evaluation target (n=8), followed by animal models (n=3). As an evaluation platform, cadaver models are a step up from anthropomorphic phantoms in terms of anatomical realism; however, they do not require the same safety critical considerations as studies with patients. Cadaver models were used in the evaluation of navigation accuracy in complex pelvic osteotomies [45], assessment of performance during immersive telementoring [58], total shoulder arthroplasty [88], maxillofacial zygomatic arch reconstruction [85], and orthopedic K-Wire placement [50]. Animal models improve on the anatomical relevance of cadaver models due to the incorporation of realistic tissue deformation and real-time respiratory and cardiac motion. Animal models were investigated for OST-HMD-led percutaneous tumor puncture [68], placement of radioactive seeds during brachytherapy [98], and media delivery in targeted cardiac therapies [33].

#### 4.6.2. System Accuracy

We have also indicated the measured accuracy of the surveyed papers in Table 6. Overall, the accuracy provided by an OST-HMD guidance system which is integrated with an existing surgical navigation suite has been demonstrated to be on the order of 2–5 mm [67,68,82,93,98,102], which is sufficient for certain surgical interventions, such as neurosurgical burr hole placement (<10 mm) [104] or complex pelvic osteotomies (<10 mm) [45]. As previously mentioned, the most popular tracking strategy for OST-HMD-camera-led surgical navigation relied on square-marker fiducials, such as ArUco [31] or Vuforia and monocular RGB video. However, the inherent assumption of planar surfaces introduced by the surface-based tracking strategy limited the precision for virtual content alignment on contoured surfaces, resulting in a tracking accuracy on the order of 5–8 mm [46,61,64,65,74]. Another source of error is the requirement for precise user-led placement of these square markers to permit accurate tracking. By leveraging the infrared cameras on the HoloLens 2 for stereo tracking, accuracy on the order of 2 mm was achieved in a phantom study [60]. Further, the introduction of an externally mounted red-green-blue-depth (RGBD) camera to the HoloLens 1 resulted in improved depth-based tracking with an accuracy of 4 mm as assessed by a phantom evaluation [77].

#### 4.6.3. Human Factors, System Usability, and Technical Challenges

**Figure 11:**
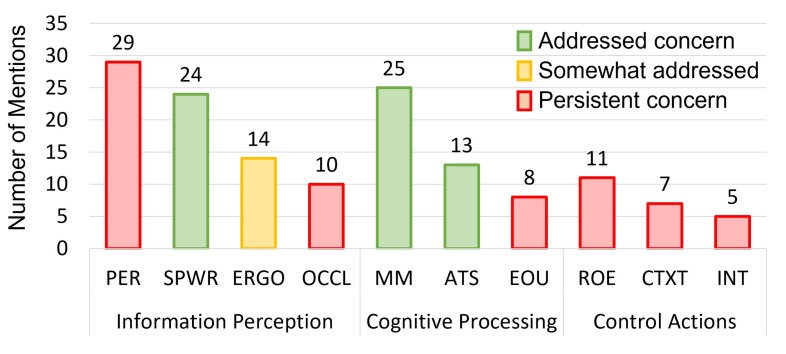
Distribution of the 57 articles included in the review based on the reported human factors related considerations. Human factors considerations included risk of error (ROE), spatial awareness (SPWR), ease of use (EOU), perception (PER), ergonomics (ERGO), attention shift (ATS), interaction challenges (INT), mental mapping (MM), context (CTXT), hand-eye coordination (HE), and occlusion (OCCL).

The final aspect of system validation which was documented in the articles was related to human factors and workflow benefits and limitations (Table 6). Human factors considerations have been consistently reported as one of the key challenges limiting the uptake of OST-HMD-based surgical navigation into the clinic [6]. In our assessment of OST-HMD-based surgical navigation systems, we categorized each reported human factors consideration as being: (1) addressed; (2) partially addressed; or (3) remaining to be addressed (persistent). As suggested by a recent FDA forum on the application of human factors and usability engineering to medical devices [105], we further categorized each individual human factors consideration into three general phases of user interaction including: (1) information perception; (2) cognitive processing; or (3) control actions. A chart summarizing the reported human factors considerations and status is included in Figure 11.

The most frequently reported persistent human factors consideration in the literature review was perception (n=29). In the context of OST-HMD use in surgery, perception refers to the quantitative assessment of a user’s interpretation and understanding of virtually augmented content. Challenges in perception of virtually augmented elements can be attributed to a number of factors, including incorrect IPD estimation [40], incorrect or unaccounted for per-user display calibration [33,97], display limitations due to vergence-accommodation [10], misregistration of virtual content due to failed registration [106], and limitations of depth understanding of virtually augmented content [107]. Perceptual limitations contributed to additional errors in the stability of calibration during ultrasound image-guided robot cervical pedicle screw placement [93], with navigation during total shoulder arthroscopy [99], and with marker-less tracking for guidance in head and neck carcinoma imaging [75].

Following perception, the next most commonly discussed human factors components were mental mapping (n=25), spatial awareness (n=24), and attention shift (n=13). In the survey, OST-HMDs were discussed as contributing to the improvement of spatial awareness and mental mapping, as well as the reduction in attention shift during surgical navigation and served to address or partially address these considerations. Spatial awareness refers to the fundamental limitation of conventional image guidance methods in their display of inherently 3D content for consumption on a 2D medium. Research into spatial awareness has indicated that 3D models of patient anatomy enable improved understanding of spatial relationships over 2D imaging slice planes [108,109]. Mental mapping is closely associated with spatial awareness and refers to the requirement for a surgeon to project (or mentally map) 2D image data onto the 3D scene in order to leverage this information for surgical guidance. A reliance on mental projection of data for surgical guidance can introduce errors to the surgery and additional mental workload on the surgeon [6,33]. Attention shift describes the requirement for a surgeon to frequently gaze between the provided navigation information on an external display and the patient; with an OST-HMD, virtual guidance information can be brought directly into the FOV of the wearer to remove this requirement. Improved spatial awareness and mental mapping as compared with traditional guidance paradigms was reported by numerous articles across different surgical applications [33,46,55,75,97]. Reduced attention shift by using an OST-HMD for the display of guidance information was measured in fluoroscopic imaging for orthopedic surgery [81] and ultrasound imaging for ultrasound-guided procedures [92].

Ergonomics (n=14) was a commonly discussed human factor consideration which was partially addressed with the use of OST-HMD led navigation. Ergonomics included physical factors, such as the effect of user posture and perceived comfort, as well as physiological factors, such as nausea and eye strain during OST-HMD use. With OST-HMD use, it is possible to position virtually augmented content in a comfortable location based on user preference. Despite the additional weight and bulk of wearing an HMD throughout a procedure, ergonomic benefits were reported in OST-HMD use for data display during robotic surgery bedside assistant tasks [56] and in live fluoroscopic imaging display during orthopedic interventions [81]. However, the contribution of OST-HMD use to ergonomics was not all positive; challenges with optical display quality and latency can contribute to nausea and eye strain [110].

Risk of error (n=11), ease of use (n=8), context (n=7), and interaction challenges (n=5) were the next most frequently discussed persistent human factor limitations. Risk of error described the concerns of potential mistakes as a result of virtual guidance. Ease of use, context and interaction collectively describe the required inputs from a user to manually adapt the current virtually augmented guidance information to be optimal for their current surgical task. Several articles discussed concerns regarding the introduction of error by relying upon virtual guidance information presented by an OST-HMD that is no longer relevant to the current anatomical scene due to loss of tracking [76] or accidental user error [61]. An additional challenge with OST-HMD led surgical guidance is the lack of shared situational awareness for the care team and staff, meaning it is solely up to the primary surgeon or user to determine the validity of aligned virtual content. Any surgical intervention can be broken down into a series of steps or phases which comprise the duration of the procedure. These phases will vary between different procedures, and during each phase there will often be different data or relevant information which are used to inform clinical decision-making. During an OST-HMD led surgical navigation procedure, if the surgeon (or a bystander) is required to manually adapt or manipulate the presented virtual content to be relevant to their current surgical context it can introduce additional ease of use and interaction challenges. Several articles mentioned workflow challenges due to the manual interaction with virtual models required to adapt the information to the current surgical task [33,69,78,83]. These workflow challenges contributed to reduced enjoyment and overall usability of the guidance system [58,86].

Occlusion (n=10), a persistent technical challenge, contributed to limitations in virtual model alignment and loss of tracking during OST-HMD based surgical navigation. With traditional navigation approaches based on an RGB or infrared camera and visible markers or retroreflective spheres, the loss of direct line-of-sight of the markers will result in loss of tracking. This is problematic as the line-of-sight requirement can be frequently interrupted over the course of an intervention due to crowding in the operating room and other surgical complications, such as blood and smoke [33,75]. By leveraging the stereo infrared cameras on the HoloLens 2 and an extended Kalman filter for pose prediction smoothing, it was demonstrated that the contribution of occlusion to loss of tracking could be reduced [60]. Further, with marker-less approaches there lies the potential to remove the requirement of the complex setup and calibration involved in traditional marker-based navigation strategies [76,99].

### 4.7. Recommendations for Future Work

Although there have been significant efforts to improve the design of OST-HMD led surgical navigation platforms, applications have remained constrained to research lab environments and there has been little clinical uptake [5,6]. As evidenced by the broad survey of recent literature, the poor clinical uptake of OST-HMDs for surgical guidance can be partially attributed to a lack of HMD performance [7], rendering resolution [8], and application or task-specific HMD design [21]; however, perceptual challenges [9,10,33], surgical context and interaction limitations [11,12], and registration and occlusion challenges [13,14] remain the key hurdles in the widespread clinical adoption of these technologies. To this end, we suggest several directions for future work which stand as meaningful initial steps in the mitigation of these limitations and in the improvement in design for more effective OST-HMD led surgical navigation platforms.

#### 4.7.1. Marker-Less Tracking for Surgical Guidance

To address a frequently mentioned limitation of current marker-based tracking solutions—occlusion and the requirement for complex setup and potential user-introduced error during marker placement—we suggest the exploration of marker-less tracking strategies for leading surgical navigation. In the context of general computer vision and AR, marker-free object pose estimation remains a challenging and unsolved problem [111]; prior strategies have utilized conventional and deep learning-based techniques [111]. Related to the surgical domain, recent work has focused on leveraging constraints imposed by typical hand-object interactions for the prediction of precise hand and rigid surgical drill pose from monocular RGB data [112] and has indicated the feasibility of marker-less surgical tracking on a commercially available OST-HMD using a data streaming approach [113].

#### 4.7.2. Context-Relevant Augmented Reality for Intelligent Guidance

Ease of use, context, and interaction challenges were repeatedly cited as limitations of current OST-HMD guidance systems and contributors to workflow and usability challenges. The requirement for a user to manually adapt the virtual information to be relevant to their current surgical context and preference can result in significant interruptions to the overall surgical workflow. These interruptions can be exacerbated by additional challenges with virtual content interaction introduced with current commercially available OST-HMDs, requiring users to perform gestures, say voice commands, or interact with virtual menus [11]. Understanding the current surgical context involves the interpretation of the vast amount of information created during a surgical procedure, which has been explored using traditional or deep learning-based [114] strategies. Recent work has explored the feasibility of OST-HMD-based guidance systems for context-relevant information display based on an interpretation of the current surgical task of the wearer of the HMD using specialized sensors [12] or monocular RGB video [115] as input.

## 5. Conclusions

In this systematic review, we discussed recent applications of OST-HMDs in AR-led surgical navigation and identified several key challenges which continue to limit clinical uptake. The most commonly reported surgical target involved orthopedic applications, likely due to the rigid nature of anatomy and availability of consistent landmarks for registration and tracking. As for input data for visualization, CT was the most common form of preoperative imaging, with fluoroscopy the most utilized form of intraoperative imaging. Preoperative CT and intraoperative fluoroscopy are commonly used in combination for leading orthopedic interventions. For visualization of imaging-derived virtual models, the most common strategy involved the display of surface rendered models or other planning information. The popularity of surface rendered models can be attributed to their ease of creation, modifiable visualization properties, and rendering performance on commercially available OST-HMDs.

The most popular overlay strategy for virtual content display involved the use of the HoloLens 1 or HoloLens 2 OST-HMDs with RGB based tracking based on ArUco or Vuforia markers. Second to this was the reliance of manual placement of virtual elements by the surgeon, followed by the incorporation of co-calibrated external tracking systems such as the Northern Digital Inc., Polaris. For interaction, a combination of voice and gesture-based or gesture-based only were the most frequently reported paradigms due to their out-of-the-box support on the HoloLens 1 and 2 devices. As for the perception location of virtual content, direct overlay of virtual content onto a target object was the most commonly pursued type of visualization.

Due to the early investigational nature of many OST-HMD based navigation platforms, an anthropomorphic phantom was used in the majority of validation studies. When integrated with an existing surgical navigation suite, accuracy on the order of 2–5 mm is feasible with an OST-HMD—a result that is sufficient for certain surgical interventions. As a stand-alone system, OST-HMDs are currently capable of accuracy on the order of 5–8 mm using marker-based tracking and monocular RGB video. In our evaluation, OST-HMD use in surgery fully or partially addressed several human factors including: mental mapping capacity, spatial awareness, attention shift, and ergonomics. However, we also identified several human factors and technical challenges commonly mentioned during OST-HMD use in surgery which continue to persist, including: perception, ease of use, context, interaction challenges, and occlusion.

Coinciding with the significant investments from leading technology companies into the metaverse and the XR technologies enabling associated shared virtual experiences, there has been increased interest in incorporating these transformational technologies, such as OST-HMDs, for improving many aspects of surgery. It is anticipated that the hardware and display limitations present in current generation OST-HMDs will be improved upon with future iterations. With additional contributions to reduce the impact of the remaining technical and human factors limitations, we believe that clinically viable OST-HMD-led surgical navigation is feasible.

## Figures and Tables

**Table 1:** Summary of technical specifications for commercially available optical see-through head-mounted displays.

Specifications	Google Glass 2	HoloLens 1	HoloLens 2	Magic Leap 1	Magic Leap 2
Optics	Beam Splitter	Waveguide	Waveguide	Waveguide	Waveguide
Resolution	640×480 px	1268×720 px	2048×1080 px	1280×960 px	1440×1760 px
Field of View	$30^{\circ }$ diagonal	$30\times 17.5^{\circ }$	$43\times 29^{\circ }$	$40\times 30^{\circ }$	$44\times 53^{\circ }$
Focal Planes	Single Fixed	Single Fixed	Single Fixed	Two Fixed	Single Fixed
Computing	On-board	On-board	On-board	External pad	External pad
SLAM	6DoF	6DoF	6DoF	6DoF	6DoF
Eye Tracking	No	No	Yes	Yes	Yes
Weight	46 g	579 g	566 g	345 g	260 g
Design	Glasses-like	Hat-like	Hat-like	Glasses-like	Glasses-like
Interaction	Touchpad	Head, hand, voice	Hand, eye, voice	Controller	Eye, controller
Release Date	2019	2016	2019	2018	2022
Price	$999	$3000	$3500	$2295	$3299
Status	Available	Discontinued	Available	Available	Upcoming

**Table 2:** Papers categorized by the data type they employed. Some articles included a combination of preoperative and intraoperative data.

Data: Preoperative or Intraoperative	Number of Articles
**Preoperative**	
Computed Tomography (CT)	34
Magnetic Resonance Imaging (MRI)	7
CT and/or MRI	6
Prerecorded Videos	1
**Intraoperative**	
Fluoroscopy	4
Ultrasound	3
Telestrations/Virtual Arrows and Annotations	3
Cone Beam CT	3
Endoscope Video	2
Patient Sensors/Monitoring Equipment	1
Simulated Intraoperative Data	1

**Table 3:** Papers categorized by the data processing type used. Some articles included a combination of processing types, such as volume and surface rendered models.

Processing	Number of Articles
**Three-Dimensional**	
Surface Models	39
Planning Information	8
Raw Data	5
Volume Models	4
Printed Models	3
**Two-Dimensional**	
Telestrations	4
Raw Data	3

**Table 4:** Papers categorized by the overlay type used. External trackers (surgical navigation suites) were used in conjunction with an optical see-through head-mounted display to co-locate the headset with relevant tracked surgical tools in frame. We indicate the frequency of commercial tracking system usage and the type of tracking marker used.

Overlay	Count	Tracking Marker	Count
**External Tracker**		**External Markers**	
Northern Digital Inc. Polaris	7	Retroreflective Spheres	11
Northern Digital Inc. EM/Aurora	3	Electromagnetic	3
ClaroNav MicronTracker	2	Visible	2
Optitrack	1		
Medtronic SteathStation	1		
**OST-HMD Camera (RGB/Infrared)**		**Optical Markers**	
HoloLens 1	19	Vuforia	10
HoloLens 2	10	ArUco	9
Custom	3	Custom	4
Magic Leap 1	1	Retroreflective Spheres	2
**OST-HMD Display Calibration**		QR-Code	2
SPAAM/similar	2	Marker-Less	2
		AprilTag	1
**Manual Placement**			
Surgeon	8		
Other	3		

**Table 5:** Papers categorized by the view type used. Interaction types included voice (VO), gesture (GE), gaze (GA), keyboard (KB), head pose (HP), pointer (PO), and controller (CNT). Display devices included the HoloLens 1 (HL1), HoloLens 2 (HL2), and Magic Leap One (ML1). Perception location included direct overlay (DO) or adjacent overlay (AO). Not applicable (N/A) methods are indicated.

View	Interaction	Display Device	Perception Location
Ackermann et al., 2021 [45]	N/A	HL1	DO
Cattari et al., 2021 [83]	N/A	Custom	DO
Condino et al., 2021 [66]	N/A	Custom	DO
Condino et al., 2021 [96]	VO, GE	HL1	DO
Dennler et al., 2021 [46]	VO, GE	HL1	AO
Dennler et al., 2021 [100]	N/A	HL1	DO
Farshad et al., 2021 [47]	VO, GE	HL2	DO
Fick et al., 2021 [63]	VO, GE	HL1	DO
Gao et al., 2021 [54]	VO	HL1	DO
Gasques et al., 2021 [58]	VO, GE, PO	HL1	DO
Gsaxner et al., 2021 [60]	N/A	HL2	DO
Gsaxner et al., 2021 [75]	GA, GE	HL2	DO
Gu et al., 2021 [99]	GA, GE	HL2	DO
Gu et al., 2021 [48]	GE	HL1	DO
Heinrich et al., 2021 [73]	VO, GE	HL1	DO
Iqbal et al., 2021 [55]	N/A	HL1	AO
Ivan et al., 2021 [62]	GE	HL1	DO
Ivanov et al., 2021 [74]	GE	HL2	DO
Johnson et al., 2021 [81]	N/A	ODG R-6	AO
Kimmel et al., 2021 [86]	VO, GE	HL1	AO
Kitagawa et al., 2021 [72]	N/A	HL2	AO
Kriechling et al., 2021 [88]	VO, GE	HL1	DO
Kriechling et al., 2021 [101]	VO, GE	HL1	DO
Kunz et al., 2021 [64]	GE	HL1	DO
Lee et al., 2021 [87]	N/A	HL2	DO
Li et al., 2021 [68]	N/A	HL1	DO
Lim et al., 2021 [80]	GE	HL2	DO
Lin et al., 2021 [57]	GE	ML1	DO
Liu et al., 2021 [102]	VO	HL1	AO
Liu et al., 2021 [103]	N/A	HL2	DO
Liu et al., 2021 [69]	N/A	HL1	DO
Majak et al., 2021 [97]	N/A	Moverio BT-200	DO
Qi et al., 2021 [65]	GE	HL2	DO
Rai et al., 2021 [59]	CNT	ML1	DO
Schlueter-Brust et al., 2021 [51]	GE	HL2	DO
Spirig et al., 2021 [50]	VO, GE	HL1	DO
Stewart et al., 2021 [56]	VO, GE	HL1	AO
Tang et al., 2021 [52]	GE	HL1	AO
Tarutani et al., 2021 [70]	GE	HL2	AO
Teatini et al., 2021 [49]	GE	HL1	DO
Tu et al., 2021 [82]	VO, GE	HL2	DO
Velazco-Garcia et al., 2021 [78]	VO, GE	HL1	AO
Yanni et al., 2021 [95]	CNT	ML1	DO
Zhou et al., 2021 [98]	VO, GE	HL1	DO
Carbone et al., 2022 [90]	N/A	Custom	DO
Doughty et al., 2022 [33]	GE	HL2	DO
Frisk et al., 2022 [84]	CNT	ML1	DO
Hu et al., 2022 [77]	KB	HL1	DO
Johnson et al., 2022 [71]	VC	HL2	DO
Ma et al., 2022 [91]	HP	HL1	DO
Nguyen et al., 2022 [92]	VO	HL1	DO
Puladi et al., 2022 [85]	GE	HL1	DO
Tu et al., 2022 [93]	GE	HL2	DO
Uhl et al., 2022 [67]	CNT	ML1	DO
Von Atzigen et al., 2022 [76]	VO	HL1	DO
Yang et al., 2022 [53]	VO, GE	HL1	DO
Zhang et al., 2022 [61]	N/A	HL1	DO

**Table 6:** Papers categorized by the validation type employed. Models for evaluation included phantom models (PHA), cadaver models (CAD), animals (ANI), and patients (PAT). Human factors considerations included risk of error (ROE), spatial awareness (SPWR), ease of use (EOU), perception (PER), ergonomics (ERGO), attention shift (ATS), interaction challenges (INT), mental mapping (MM), context (CTXT), hand-eye coordination (HE), and occlusion (OCCL). Not applicable (N/A) methods are indicated.

Validation	Evaluation	Accuracy	Human Factors
Ackermann et al., 2021 [45]	CAD	10.8 mm RMS, ${\left(6.7,7.0,0.9\right)}^{\circ }$	ROE, SPWR, EOU
Cattari et al., 2021 [83]	PHA	2.02 mm	PER, ERGO, ATS, MM, CTXT
Condino et al., 2021 [66]	PHA	1.3±0.6 mm	ATS, PER, SPWR, CTXT, INT
Condino et al., 2021 [96]	PHA, PAT	N/A	SPWR, MM, PER
Dennler et al., 2021 [46]	PAT	N/A	ERGO, SPWR, ATS, PER
Dennler et al., 2021 [100]	PHA	7.3±1.9 mm entry, $11.9\pm 3.1^{\circ }$	ATS, ROE
Farshad et al., 2021 [47]	PAT	3.5±1.9 mm entry, $11.9\pm 3.1^{\circ }$	ATS, ROE, ERGO, PER
Fick et al., 2021 [63]	PAT	8.5 mm	MM, ATS, SPWR
Gao et al., 2021 [54]	PHA	1.036±0.081	SPWR, MM, PER
Gasques et al., 2021 [58]	PHA, CAD	N/A	ROE, PER, CTXT
Gsaxner et al., 2021 [60]	PHA	1.90 mm $1.18^{\circ }$ RMS	PER, INT, CTXT, SPWR
Gsaxner et al., 2021 [75]	PHA	N/A	EOU, PER, MM
Gu et al., 2021 [99]	PHA	3.80±1.28 mm, $4.66\pm 2.85^{\circ }$	PER, MM, SPWR, OCCL, CTXT
Gu et al., 2021 [48]	PHA	4.87±2.97 mm, $5.95\pm 2.01^{\circ }$	OCCL, PER
Heinrich et al., 2021 [73]	PHA	N/A	PER, HE
Iqbal et al., 2021 [55]	PAT	Surface Roughness	EOU, ERGO, PER, MM, SPWR
Ivan et al., 2021 [62]	PAT	Trace Overlap	ERGO, SPWR
Ivanov et al., 2021 [74]	PAT	3−7 mm	MM, PER
Johnson et al., 2021 [81]	PHA	N/A	ERGO, EOU
Kimmel et al., 2021 [86]	PAT	N/A	CTXT
Kitagawa et al., 2021 [72]	PAT	N/A	SPWR, EOU
Kriechling et al., 2021 [88]	CAD	3.5±1.7 mm $3.8\pm 1.7^{\circ }$	N/A
Kriechling et al., 2021 [101]	CAD	2.3±1.1 mm $2.7\pm 1.31^{\circ }$	N/A
Kunz et al., 2021 [64]	PHA	4.8±2.5 mm	CTXT, PER, ERGO
Lee et al., 2021 [87]	PHA	N/A	PER, INT, SPWR
Li et al., 2021 [68]	PHA, ANI	1.68 mm	PER, INT, SPWR
Lim et al., 2021 [80]	PHA	N/A	N/A
Lin et al., 2021 [57]	PHA	4.67 mm	CTXT
Liu et al., 2021 [102]	PAT	1.441±0.234 mm	SPWR, MM
Liu et al., 2021 [103]	PAT	Radiation Exposure	ERGO, EOU
Liu et al., 2021 [69]	PHA	3±1 mm	CTXT, PER
Majak et al., 2021 [97]	PHA	2.34±0.88 mm	MM, ATS, SPWR
Qi et al., 2021 [65]	PAT	5.4±0.9 mm	MM, SPWR, ROE
Rai et al., 2021 [59]	PAT	N/A	SPWR, EOU
Schlueter-Brust et al., 2021 [51]	PHA	3 mm $5^{\circ }$	OCCL, PER
Spirig et al., 2021 [50]	CAD	5.99±3.6 mm $5.88\pm 3.69^{\circ }$	MM, ATS, SPWR
Stewart et al., 2021 [56]	PHA	N/A	ATS, ERGO
Tang et al., 2021 [52]	PAT	N/A	HE, SPWR, MM, PER
Tarutani et al., 2021 [70]	PHA	1.67 mm	ROE, SPWR
Teatini et al., 2021 [49]	PHA	8.22±2.27 mm	SPWR, MM, PER, HE, ROE
Tu et al., 2021 [82]	PHA, CAD	6.1±1.45 mm	MM, OCCL, HE, PER, ERGO
Velazco-Garcia et al., 2021 [78]	PHA	N/A	MM, CTXT, SPWR
Yanni et al., 2021 [95]	PHA	N/A	ERGO, MM, PER
Zhou et al., 2021 [98]	PHA, ANI	1.586 mm $2.429^{\circ }$	OCCL, INT, ROE
Carbone et al., 2021 [90]	PHA, PAT	±1 mm	ROE, OCCL, PER, ERGO
Doughty et al., 2022 [33]	PHA, ANI	0.98±0.5 mm	PER, MM, OCCL, CTXT, ATS
Frisk et al., 2022 [84]	PHA	1.9±0.7 mm $3.0\pm 1.4^{\circ }$	MM, ATS
Hu et al., 2022 [77]	PHA	4.36±0.8 mm $5.65\pm 1.42^{\circ }$	OCCL, PER
Johnson et al., 2022 [71]	PHA	3.0±1.5 mm	PER, MM, ERGO
Ma et al., 2022 [91]	PHA	N/A	OCCL, ERGO, EOU
Nguyen et al., 2022 [92]	PHA	N/A	HE, MM
Puladi et al., 2022 [85]	CAD	2 mm	MM, SPWR, PER, OCCL
Tu et al., 2022 [93]	PHA	1.04±0.27 mm	MM, HE, PER, ROE
Uhl et al., 2022 [67]	PHA	2.1 mm	MM, ATS
Von Atzigen et al., 2022 [76]	PHA	5.43 mm	ATS
Yang et al., 2022 [53]	PAT	5.54 mm	SPWR, MM
Zhang et al., 2022 [61]	CAD	4.21±1.6 mm	MM, ROE

## Data Availability

Additional literature search data are available on request from the corresponding author.
